# Prof. Smith’s Paper

**Published:** 1894-08

**Authors:** 


PROF. SMITH'S PAPER, in this issue, is as timely as it is able. It will be read with much interest, as embodying the views of one who speaks by the book. It is unfortunate that it can not reach and be read by all the people, for it is high time they were being made aware that, although consumption is highly contagious, it is the easiest matter in the world to prevent its spread. It is now definitely known that the sputa is the danger element, and that the mode of propagation is through its becoming dried—when the infective bacilli float in the air and find lodgment in the air passages of those who breathe it. Destroy the sputa—avoid kissing and sleeping with a consumptive—observe all rules of cleanliness and decency in and about the apartments occupied by the patient, and the disease will be practically disarmed of its dangers.

We commend the paper to the careful perusal of our readers.

